# Fluorescence based real time monitoring of fouling in process chromatography

**DOI:** 10.1038/srep45640

**Published:** 2017-03-30

**Authors:** Mili Pathak, Katherine Lintern, Viki Chopda, Daniel G. Bracewell, Anurag S. Rathore

**Affiliations:** 1 Department of Chemical Engineering, Indian Institute of Technology, New Delhi, India; 2 Department of Biochemical Engineering, University College London, Torrington Place, London, UK

## Abstract

A real time monitoring of fouling in liquid chromatography has been presented. The versatility of the approach has been proven by successful implementation in three case studies with an error <1%. The first application demonstrates the monitoring of protein A ligand density and foulant concentration for assessing performance of protein A chromatography resin during purification of monoclonal antibodies. The observations have been supported from LC-MS/MS studies that were independently performed. The second application involves monitoring of foulant deposition during multimode cation exchange chromatography based purification of human serum albumin. Finally, in the third application, monitoring of foulants during multimodal hydrophobic interaction chromatography of recombinant human granulocyte colony stimulating factor is demonstrated. In all three cases, it is observed that the fluorescence intensity consistently increases with resin reuse as more foulants are deposited over time. The proposed approach can be readily used for real time monitoring of fouling and process control.

Chromatographic separation continues to be the mainstay for purification of biotherapeutic products. Although ubiquitous in biopharmaceutical processes, chromatographic steps are major contributors to the overall cost of raw material in the manufacture of biotherapeutic products. In order to improve process economics, it is common practice to reuse chromatography resins. Resin reuse, however, is known to impact product yield and in some cases even product quality. This is particularly true in the case of protein A chromatography, the capture step used for the purification of monoclonal antibody and Fc fusion protein products^1^,^2^. For optimal operation, it is critical that the performance of the process chromatography column to be assessed during processing and appropriate steps such as cleaning and repacking taken to ensure that the product quality and yield are consistently maintained throughout the resin lifecycle^3^,^4^. Resin fouling is a complex problem that can be influenced by several factors such as impurities or residual product deposition on pores which may either block pores and/or block binding sites on the surface of resin. Interactions between the deposited materials and/or with different conditions or steps such as cleaning protocols also influence resin fouling^5^. Current best practice is to use a combination of holistic indicators to monitor resin fouling. These indicators include measurements of uniformity of column packing (plate height, asymmetry), product yield, and product quality. This approach suffers from the fact that none of these indicators are a direct measure of resin fouling and consequently a significant amount of uncertainty is introduced in the assessment of column performance. An approach that allows for direct, quantitative assessment of fouling would be of great interest to the biopharmaceutical industry.

Researchers have attempted to monitor deposited material on resins via microscopy. This approach is, however, time consuming and preparation of the resin sample for microscopy requires expertise. Furthermore, collection of the sample from the process column during manufacture is not feasible. In contrast, fluorescence spectroscopy is particularly well suited to monitor fouling of chromatography resin. Unlike other analytical tools such as circular dichroism, dynamic light scattering, or mass spectrometry, fluorescence spectroscopy can readily probe heterogeneous samples such as agarose beads. Researchers have demonstrated ATR-FTIR as a tool to monitor affinity chromatography purification of monoclonal antibody, but the measurement requires repacking of the resin and so cannot be directly applied to perform continuous monitoring of process scale chromatography^6^.

In this communication, we propose a robust fluorescence based approach to monitor fouling in process chromatography resin by presenting its use in three independent studies^7^. The versatility of the approach has been demonstrated by successful implementation in three case studies that involve use of three different modes of process chromatography for purification of three different biotherapeutic products. In view of the results, it is evident that the proposed approach can be readily used for real time monitoring of fouling and for process control.

## Results

**Figure 1:**
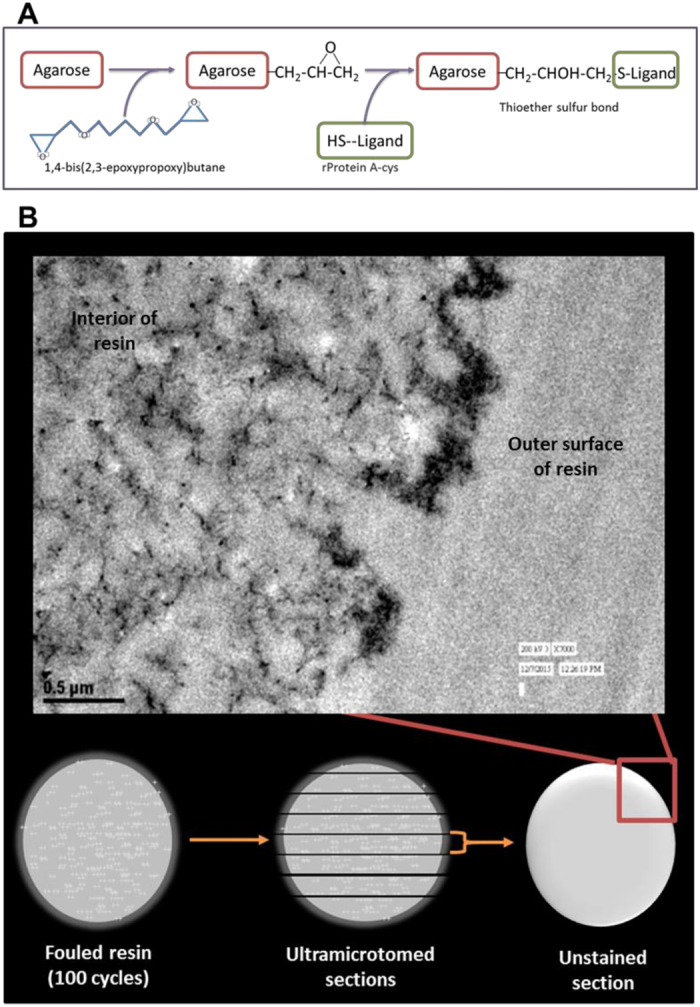
(**A**) MabSelect resin chemistry. (**B**) TEM image for resin samples obtained after 100 cycles of reuse. The TEM image shows fouling of the chromatography resin.

Protein A chromatography is the default process step for capture of antibody products^8^, typically comprising of functionalized protein A ligand attached to a highly crosslinked agarose bead through an epoxy linkage^9^ (Fig. 1A). Resin performance loss could be due to ligand leaching, deposition of foulant on the resin surface and pores, and/or irreversible binding of mAbs^10^. Figure 1B presents a transmission electron microscopy (TEM) image of a fouled resin sample after 100 cycles. TEM was performed after dehydrating the resin samples and washing them with increasing ethanol concentrations from 0 to 100% anhydrous ethanol. Sample was then embedded in LR-White resin and ultramicrotomed into sections and was viewed unstained^11^. It is observed that the fouled resin sample from the 100^th^ cycle exhibits deposition of foulants with a film thickness of 0.3 μm (Fig. 1B). After the 100^th^ cycle, the chromatographic yield was calculated to be ~60% with a loss in binding capacity of 80%. The major cause of fouling in this case was the non-specific adsorption of the feed material components on the resin^10^.

Direct measurement of the foulants present on resin can facilitate the timely use of appropriate cleaning conditions to clear the deposits before a detrimental effect is observed. Otherwise, with increasing time, the deposited foulants may continue to build-up on the resin surface and eventually require use of very harsh conditions to clean, which would likely cause irreversible damage to the resin ligands.

**Figure 2:**
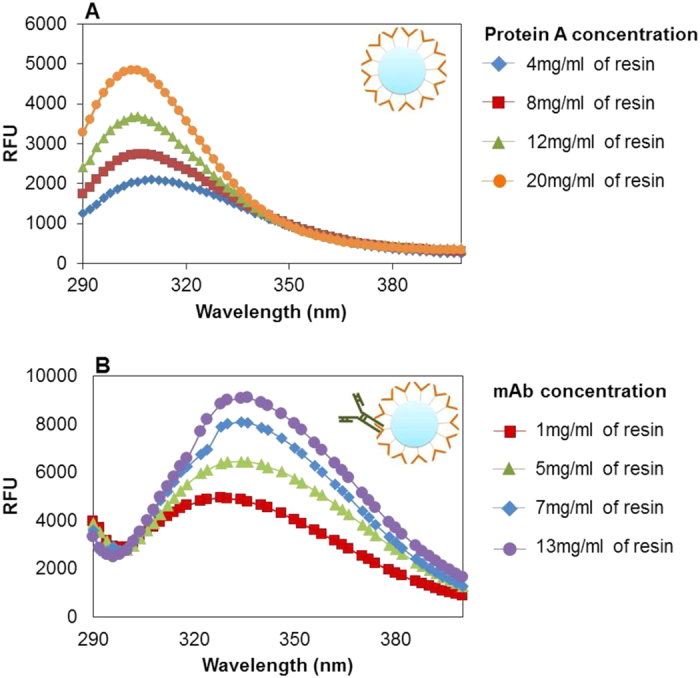
Fluorescence emission spectra (290–400 nm) for (**A**) different concentrations of protein A ligand present on the resin interior and exterior surface, and (**B**) different concentrations of mAb bound to protein A ligand present on the resin. Images top right of each pane are stylized representations of fresh MabSelect SuRe^TM^ resin and the MabSelect SuRe^TM^ resin bound to mAb. For the latter case, fixed volume of MabSelect SuRe^TM^ resin was incubated with increasing volume of mAb solution (to increase IgG concentration) and the unbound mAb was removed by given a wash with PBS. The resulting resin samples with the mAb bound to protein A resin were examined via fluorescence.

Recombinant protein A contains tyrosine and phenylalanine residues but lacks tryptophan. In contrast, tryptophan is present in common foulants (host cell proteins and mAb^12^), along with tyrosine and phenylalanine. Thus, the fluorescence intensity at 340 nm (Lambda max for tryptophan) is primarily due to foulants and mAb present on the resin, while the fluorescence intensity at 303 nm (Lambda max for tyrosine) is due to the protein A ligand present on the resin as this is the dominant species. This was verified by measuring the fluorescence intensity of fresh MabSelect SuRe^TM^ resin samples together with MabSelect SuRe^TM^ resin samples bound with varying concentrations of mAb (Fig. 2A and B). The concentration of protein A ligand present on the MabSelect SuRe^TM^ resin was estimated by calculating the DBC_10%_ (mg.ml^−1^) for fresh resin, as one attached protein A ligand can bind up to two mAb molecules^13^. To estimate the lambda maxima for protein A ligand, fresh MabSelect SuRe^TM^ resin was analyzed with the fluorescence tool in the offline mode using different slurry percentages (resin slurry was prepared in PBS buffer) to achieve different protein A ligand concentrations. Figure 2A represents the fluorescence spectra at different concentrations of protein A ligand. It is observed that the intensity at 303 nm increases with the respective increase in protein A ligand concentration. To understand the change in the lambda maxima and the change of intensity at 303 and 340 nm upon foulant deposition, a fixed amount of MabSelect SuRe^TM^ resin slurry was incubated for 10 minutes with different concentration of purified mAb (IgG1) in PBS buffer pH 7.2. After incubation, the resin slurry was washed 5 times with an interval of 15 minutes to remove any unbound mAb and fluorescence analysis was performed. It was observed that as concentration of mAb bound to the chromatography column increases, the fluorescence intensity at 340 nm increases proportionally while the intensity at 303 nm remains constant (Fig. 2B). This may be because, the tyrosine fluorescence for the mAb molecules has been observed to be quenched by the presence of nearby tryptophan moieties either through resonance energy transfer or the ionization of its aromatic hydroxyl group^14^. This suggests that the proposed approach can be effectively used for monitoring both the degradation/leaching of the protein A ligand as well as the deposition of foulants on the resin surface.

**Figure 3:**
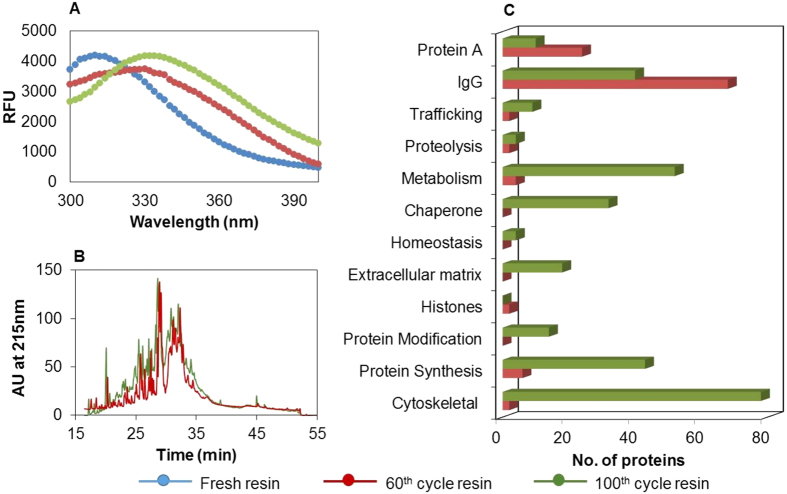
(**A**) Emission spectra (300–400 nm) for fresh and fouled resins (60th and 100th cycles). (**B**) RP-HPLC chromatogram for digests of resins cycled 60 and 100 times. (**C**) Number of different proteins present on the surface of the fouled resin sample after 60 and 100 cycles as inferred from LC-MS/MS.

The proposed approach was applied for monitoring fouling of protein A resin during purification of a monoclonal antibody therapeutic. Figure 3A represents the difference in the emission spectra for fresh and fouled protein A resin used for purification of IgG1 from clarified cell culture broth (CCCB). It is observed that as the resin becomes more fouled, the maxima in the relative fluorescence unit (RFU) vs. wavelength plot shifts from 303 nm to 340 nm. This indicates a gradual decrease in the density of the protein A ligand as well as accumulation of foulants on the resin surface with the increasing number of reuses.

These observations were confirmed by LC-MS/MS analysis of the fouled resin sample^12^. The peptide masses from this analysis with an ion score exceeding the threshold set for p < 0.05 were investigated using the Mascot algorithm (matrixscience.com) to search all taxonomies in the SwissProt database. In order to facilitate the identification and interpretation of any trends in abundance and profile of host cell proteins (HCPs), proteins were grouped according to their published UniProt primary *in vivo* role. Figure 3B represents the RP-HPLC chromatogram for the resin digest utilized for purification of the IgG1 molecule. It is observed that the peak area for the 100^th^ cycle is greater than that of the 60^th^ cycle by 19%. The relative proportion of protein A ligand present on resin at the end of 100^th^ cycle was less than that present at the end of 60^th^ cycle. This observation is in agreement with the decrease in protein A ligand intensity and increase in HCP deposition on resin upon reuse that has been reported in literature^12^. In yet another publication, researchers have reported that there is no significant leaching of protein A ligand during elution as the eluate samples have very low levels of detectable protein A leachates, and hypothesized that leaching could be during cleaning^6^. Furthermore, it is shown in Fig. 3C that the number of proteins identified from their peptides detected by LC-MS/MS present on the agarose resin were 116 and 304 at the end of 60^th^ and 100^th^ cycles, respectively. The LC-MS/MS results are found to be in agreement with the fluorescence data for the fresh and fouled resin samples, in that the accumulation of foulant increases with the number of resin cycles.

**Figure 4:**
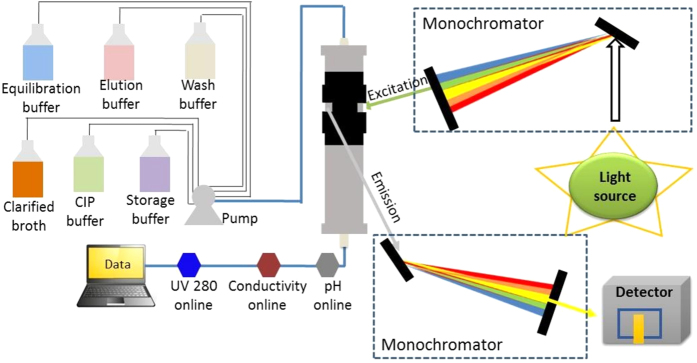
Illustration of the proposed experimental setup for real time monitoring of foulant deposition during resin reuse.

**Figure 5:**
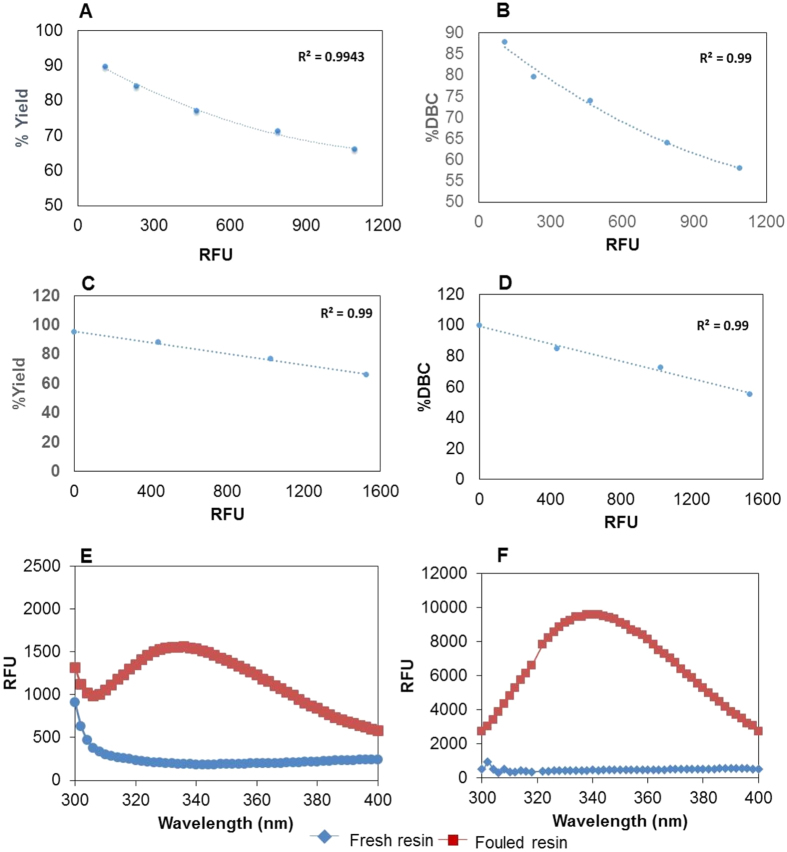
Relationship between % yield and fluorescence intensity (at 340 nm) with protein A chromatographic reuse for IgG 4 (**A**) and IgG 1 (**C**). Relationship between % DBC and fluorescence intensity (at 340 nm) with protein A chromatographic reuse for IgG 4 (**B**) and IgG 1 (**D**). Case studies for monitoring resin fouling during (**E**) multimode cation exchange chromatography for HSA purification and (**F**) multimodal hydrophobic interaction chromatography for GCSF purification.

Figure 4A illustrates a novel process control scheme where real time monitoring of fouling in process chromatography is performed and implemented for process control. Each chromatography cycle consists of sequential steps including pre-equilibration, equilibration, sample loading, wash (selective/non-selective), elution, regeneration (stripping), cleaning and wash. The equipment that is typically used to perform process chromatography incorporates online monitoring tools for UV absorbance, conductivity, and pH probes to monitor the flow-stream at the outlet of the column. We have proposed a modification to such a system by adding a fluorescence module that would facilitate real-time monitoring of column fouling. The fluorescence intensity is recorded and the deposition of foulants is estimated by subtracting the fluorescence spectra for the freshly packed column. The increase in the intensity at the end of each cycle can be then used to estimate deposition of foulants on the resin over reuse. Figure 5A–D represent the results from protein A chromatography cycling study with IgG 4 (Fig. 5A and B) and IgG 1 (Fig. 5C and D), respectively. The plot of fluorescence intensity vs. % yield was found to fit an empirical relationship (polynomial fit for Fig. 5A and B and linear fit for Fig. 5C and D) with an R_2_ value of 0.99.

**Figure 6:**
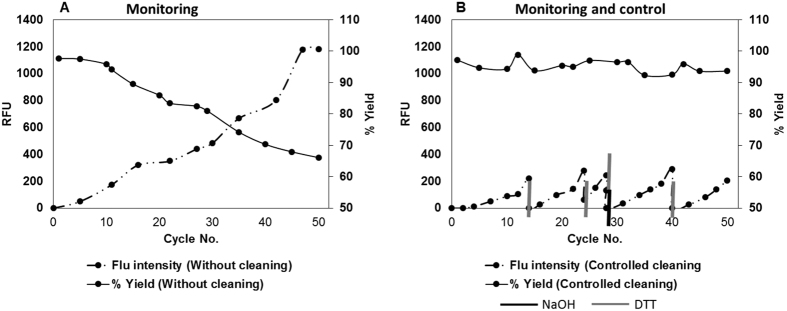
(**A**) Graph showing real time monitoring data of protein A chromatography resin reuse. It is seen that there is a consistent decrease in yield and increase in the intensity of the fluorescence signal with increasing number of resin reuses. (**B**) Graph showing real time monitoring and control data of protein A chromatography resin reuse. Cleaning with DTT and NaOH is as indicated in the Figure. It is seen that online monitoring of resin fouling allows us to the take the necessary and timely action thereby minimizing the loss in column yield.

A case study using this method to monitor and control the fouling of protein A chromatography was performed. Initially the fouling of protein A chromatography resin was monitored without providing intermediate cleaning. The yield was found to decrease with the increase in fluorescence intensity as shown in Fig. 6A. It was observed that once the fluorescence intensity exceeded 300 RFU the yield decreased below 90%. Figure 6B presents the data obtained when protein A chromatography fouling was monitored and the process controlled to avoid performance loss. The fluorescence intensity was monitored every two cycles and when the fluorescence intensity increased above 200 RFU (excitation at 280 nm and emission at 340 nm) cleaning was performed with DTT/DTT followed by NaOH with a contact time of fifteen minutes. Cleaning regimes were established in prior studies. The cleaning regime, including reagent and contact time, was decided by the user, based on their experiences. A cleaning protocol consisting of DTT followed by NaOH was selected as it resulted in maximum foulant clearance (>90%) from resin with negligible ligand leaching (<1%). This finding is supported with DBC_10%_ measurement for 0th, 50th and 200th cycles (100%, 97% and 98%, respectively). Thus for the second case, the yield was greater than 90% with the fluorescence intensity of less than 300 RFU (Fig. 6B). These results demonstrate that the developed online real-time monitoring tool could be utilized to monitor and control chromatography performance loss.

To examine the versatility of the proposed approach, it was utilized for two other protein purification applications. In the first application, fouling was monitored in the multi-mode cation exchange resin (Capto MMC) for purification of recombinant human serum albumin (rHSA) produced in *Pichia pastoris*. The chromatographic resin utilized consists of a highly crosslinked agarose matrix with a multimodal weak cation exchanger. The fresh resin did not show any emission in the range of 300 to 400 nm while the fouled resin did (Fig. 5E). The results support the efficacy of the proposed fluorescence based approach for real time monitoring of deposition of foulants (foulants in this case consist of primarily host cell proteins and the irreversibly bound HSA).

The third application involved monitoring the fouling of multimodal hydrophobic interaction chromatography (HEA Hypercel) resin during purification of recombinant human granulocyte colony stimulating factor (rGCSF). This resin consists of a highly cross-linked cellulose matrix with hexylamine and phenylpropanolamine synthetic ligands. Once again, it was observed that while the fresh resin did not exhibit any emission in the range of 300 to 400 nm (Fig. 5F), the fouled resin does, thereby indicating deposition of foulants (foulants in this case primarily consist of host cell proteins and the irreversibly bound rGCSF).

## Discussion

This work proposes a novel fluorescence approach for real time monitoring of fouling in chromatography columns. The efficacy of the approach has been demonstrated for three different applications. The proposed approach can be used for development of cleaning and sanitization regime for analytical and process chromatography, for real time monitoring of fouling, and for control of column operations and making decisions on when and how to clean and/or repack the column. The proposed approach addresses a considerable limitation in process understanding and control in biomolecule manufacture. Its implementation will certainly assist manufacturers in the development of more robust and economical downstream manufacturing processes for purification of biotherapeutic products.

## Methods

### TEM imaging

Transmission electron microscopy (TEM) was performed after dehydrating the resin samples and washing them with increasing ethanol concentrations from 0 to 100% anhydrous ethanol. Each sample was then embedded in LR-White resin and ultramicrotomed into sections. For better contrast, sections of the virgin resin samples were stained with a mixture of uranyl acetate and lead citrate, while sections of the cycled samples were viewed unstained^11^.

### Estimation of Dynamic Binding Capacity (DBC)

DBC at 10% of the breakthrough curve was determined on the fresh resin (before starting cycling studies on the column) and in between cycling studies using purified mAb. The calculation for calculating the dynamic binding capacity is given in the following equation:


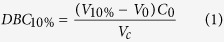


where, *C*_*0*_ = antibody concentration (mg/ml), *V*_*c*_ = geometric total volume (ml), and *V*_*0*_ = void volume (ml).

### LC-MS/MS analysis

For this analysis 50 μl resin (100 μl 50% slurry) was suspended in 50 μl 8 M urea, 100 mM ammonium carbonate. Next, 2 μl of 450 mM dithiothreitol was added and the mixture incubated at room temperature for one hour. This was followed by incubation with 20 μl of 100 mM iodoacetamide for 15 minutes at room temperature to achieve carbidomethylation of the sample. Urea was diluted by the addition of 128 μl MilliQ water prior to incubation at 37˚C for 1 hour with 5 μg modified sequence grade trypsin (Promega, Southampton, UK). Samples were applied to a C18 Acclaim PepMap100 column (75 μm x 15 cm) for reverse phase HPLC (Nano LC Ultimate3000). Elution was performed with a linear acetonitrile gradient (solvent A = 0.05% TFA, solvent B = 0.05% TFA, 90% acetonitrile) in a 40 minute cycle at a flow rate of 300 nl/min. LC was coupled to a fraction collector (Proteineer fcII, Bruker), dividing eluates into 120 fractions and mixing with matrix solution (α-cyano-4-hydroxycinnamic acid, reconstituted according to manufacturer’s guidelines in 89% acetonitrile, 0.1% TFA) prior to being spotted onto a MALDI-TOF target plate (MTP AnchorChip600 384 T F, Bruker). MALDI-TOF-TOF was conducted using an UltrafleXtreme MALDI-TOF instrument (Bruker) in positive ion reflector mode and 50% laser power and MS-MS was conducted on the ten most intense peaks for each target spot. Generated peptide masses with an ion score exceeding the threshold set for p < 0.05 were investigated using the Mascot algorithm (matrixscience.com) to search all taxonomies in the SwissProt database. Search parameters were 547599 sequences analyzed in the selected database, fixed modifications: carbidomethyl (C); variable modifications: oxidation (M); mass values: monoisotopic; protein mass: unrestricted; peptide mass tolerance:+/−50 ppm; fragment mass tolerance: + /− 0.5 Da; instrument: MALDI-TOF-TOF^12^.

### Resin cycling studies

Protein A chromatography purification was performed on a 3 ml column of ~15 cm bed height (MabSelect SuRe^TM^ from GE Healthcare Life Sciences, Stockholm, Sweden) using an Äkta Purifier system (GE Healthcare Life Sciences, Stockholm, Sweden). Within each purification cycle, columns were equilibrated with 25 mM phosphate, 50 mM NaCl, pH 6.2 buffer, for a minimum of 5 CV. Feed material consisting of clarified cell culture broth (CCCB) was then loaded on to the protein A column at a protein load of 15 mg/ml^−1^ of resin. The product was eluted from the column using 100 mM acetate, pH 3.5 buffer. The column was then regenerated with 2 M NaCl and cleaned with 50 mM NaOH, 1 M NaCl (contact time less than 15 minutes). The mobile phase velocity used was 200 cm/h^−1^. During this study, cleaning was performed after every three cycles.

Prepacked 1 ml, hexylamine aromatic (HEA) Hypercel column was used for purification of rGCSF from refolded protein mixture (5 mm ID x 50 mm bed height (Pall Life Sciences, USA)). Five column volumes of equilibration buffer were used to equilibrate the chromatography column before using. The protein loading concentration was kept constant at 5 mg for all the experiments. Acetate buffer (35 mM with pH 5.7 with 350 mM NaCl) was used to load protein onto the column. Acetate buffer (100 mM, pH 4.7) and citrate buffer,(100 mM, pH 3), were used for elution. Elution was performed with a linear gradient of 30 CV with the acetate buffer (with decreasing conductivity and pH) followed by a step gradient of 10 CV with the citrate buffer.

Purification of rHSA produced in *Pichia pastoris X-33* Mut^+^ strain, were carried out on Capto MMC multimode chromatography. Chromatographic purification step was scaled down to 1 ml column. The column was equilibrated with 25 mM acetate buffer, 10 mM NaCl, pH 5.3 ± 0.2. This was followed by loading of the clarified cell culture broth (CCCB) at a loading capacity of 5 mg/ml−1 of resin. Post loading, a minimum of five CV of equilibration buffer was passed through the column. Elution was performed using 15 mM sodium phosphate, 300 mM NaCl, pH 7.5 ± 0.2 buffer, and was followed by regeneration with 1 M NaCl and cleaning with 100 mM NaOH. During purification the residence time of 5 minutes was maintained throughout the process.

### Online monitoring setup

Referring to Fig. 4, a method for online fluorescence monitoring of foulants setup comprises of a column and different buffer containers. The fluorescence measurement setup comprises a light source, an excitation monochromator, a slit and a polarizer, a column chamber, an emission monochromator and a detector. The column has a column holder which consists of the black sheet surrounding the column with the specified window space to detect the foulants present on the resin. The holder is adapted to measure the foulants present on the chromatographic column and is made of polyoxymethylene. The sample chamber is designed to measure the foulants present on the chromatographic column with a path length of 1 cm, bandwidth (EX, EM) of 9, 9 nm, and a slit width of 5 nm. The light source is Xenon light.

The method comprises packing of a column with fresh chromatographic resin. In a next step, the packed column is subjected to a first wash with the wash buffer followed by a second wash step with equilibration buffer. The packed column is then subjected to a third wash using the wash buffer, after which the initial fluorescence intensity is recorded.

Next, equilibration buffer is passed through the column, followed by passing the clarified broth through the column. In a next step, the column is subjected to wash with the wash buffer followed by elution. In a next step, CIP buffer and/or regeneration buffer is passed through column. The column is again washed with wash buffer. The column is stored by passing storage buffer through it when not in use. Said storage buffer is removed while using it next time and the resin is further washed and equilibrated using the wash buffer and equilibration buffer. The equilibration, sample loading, washing and elution are repeated in every cycle.

The monitoring of foulant deposition on the resin during initial fluorescence intensity recording of fresh resin, recording after every cycle is described in detail hereinafter: The column excitation and emission is recorded at 250 nm to 500 nm as a spectra or a single point reading for fresh resin. In a final step, the foulant deposition on the resin over cycling is monitored by the fluorescence measurement setup as shown in Fig. 4. The light emitted by the light source is transferred to the slit and polarizer through the excitation monochromator. The slit and polarizer transmits light of a predefined wavelength to the emission monochromator. The predefined wavelength emitted by the emission monochromator is passed through the black sheet of the column packed with resin thereby resulting in the recording of fluorescence intensity by the detector. Specifically, the fluorescence intensity is recorded at every cycle and the deposition of foulants is estimated by subtracting the reading obtained for the freshly packed column with fresh chromatographic resin. The increase in the amount of fluorescence intensity at the end of respective cycles estimated the deposition of foulants on the resin over reuse.

## Additional Information

**How to cite this article**: Pathak, M. *et al*. Fluorescence based real time monitoring of fouling in process chromatography. *Sci. Rep.* **7**, 45640; doi: 10.1038/srep45640 (2017).

**Publisher's note:** Springer Nature remains neutral with regard to jurisdictional claims in published maps and institutional affiliations.
